# First Insight on the Mucus of the Annelid *Myxicola infundibulum* (Polychaeta, Sabellidae) as a Potential Prospect for Drug Discovery

**DOI:** 10.3390/md17070396

**Published:** 2019-07-05

**Authors:** Loredana Stabili, Margherita Licciano, Adriana Giangrande, Carmela Gerardi, Sandra Angelica De Pascali, Francesco Paolo Fanizzi

**Affiliations:** 1 Istituto di Ricerca sulle Acque, Sede Secondaria Talassografico di Taranto, CNR, Via Roma 3, 74123 Taranto, Italy; 2 Dipartimento di Scienze e Tecnologie Biologiche ed Ambientali, Università del Salento, Via Prov.le Lecce Monteroni, 73100 Lecce, Italy; 3 Istituto di Scienze delle Produzioni Alimentari, U.O.S. di Lecce, via Prov.le Lecce-Monteroni, 73100 Lecce, Italy

**Keywords:** mucus, Polychaeta, NMR spectroscopy, antioxidant activity, antibacterial activity

## Abstract

Many marine organisms, including invertebrates, produce mucosal matrices having different functions. Besides mechanical protection, the mucus of many invertebrates contains specific compounds to make the animal poisonous and/or distasteful or irritating. The presence of antibiotic molecules is more advantageous for some invertebrates to contrast bacterial attack. In the present study we investigated the mucus of the Mediterranean annelid species *Myxicola infundibulum* living in a gelatinous envelope made up of dense mucus. Antimicrobial lysozyme-like and antioxidant activities were investigated to highlight the potential interest of the worm mucus as a source of bioactive compounds for biotechnological applications. In order to understand which kind of compounds could be responsible for the detected activities, the mucus of *M. infundibulum* was chemically characterized in terms of elemental composition, protein, lipid and carbohydrate content. Further chemical characterization was achieved by the advanced analytical technique of multinuclear and multidimensional NMR spectroscopy. NMR spectroscopy revealed the scarcity of lipids which preferentially resulted of alcoholic origin, or otherwise hydroxylate and several aminoacids (valine, leucine and alanine) in the aqueous extract in relation to the protein nature of *M. infundibulum* mucus. The mucus indeed is mainly composed by water (94% ± 0.7%) whereas its dry weight is made of proteins (36% ± 2.3%) followed by lipids (2.9% ± 0.07%) and carbohydrates (2% ± 0.31%). The mucus exerted a natural antibacterial lysozyme-like activity corresponding to 1.14 mg mL^−1^ of hen egg-white lysozyme and an antioxidant activity corresponding to 483.00 ± 79.22 nmolTE (Trolox equivalent)/mL sample as Trolox Equivalent Antioxidant Capacity (TEAC) and 276.26 ± 50.76 nmolTE/mL sample as Oxygen Radical Absorbance Capacity (ORAC). Therefore, our findings have potential implications due to the ongoing explosion of antibiotic resistant infections and the need to discover antibacterial agents. Additionally, the observed antioxidant activity is intriguing taking into account the need to find natural antioxidants useful for human health.

## 1. Introduction

Several marine organisms release mucosal matrices showing diverse properties, in relation to the composition, structure, and evolutionary function. The mucus is a complex aqueous fluid with a high percentage of water (above 95%) and consists of a network of polysaccharides and proteins entangled to form a weak gel. In marine invertebrates, mucus has a large range of attributes and functions and its biophysical characteristics related to its function, i.e., the capability to form gels of different elasticity and viscosity, are dependent on a particular category of glycoproteins, known as mucins. Mucins are particularly large glycoproteins (0.5–2.0 MDa) characterized by the presence of a central protein core with heavily glycosylated side-chains. The levels of glycosylation differ, as the post-translational modification of glycosylation is not an exact process, however the carbohydrate level of mucins is usually around 80% [1]. 

In several marine invertebrates a mucus layer furnishes a physical shield [2] and typically forms a slippery external surface coating that performs several specific roles. In addition to mechanical protection, the mucus of many invertebrates contains specific molecules capable to render the animal distasteful, poisonous or irritating, or a mixture of these features [3]. Calow [4] suggested that mucus could be more or less vulnerable to microbial attack. Some invertebrates lace their mucus with antibiotics if it is more beneficial for them to hinder bacterial attack; in this case the mucus has low protein content and does not support bacterial growth. By contrast, some invertebrates may secrete mucus with high content of proteins rapidly employed by microorganisms [5,6]. 

Within annelids, mucus produced by polychaetes constitutes a key factor shaping the capability of many species to survive in their environment [7]. Mucus secretion is very widespread among this invertebrate group, and this is correlated with its multiple functions. It is involved in egg fertilization and first larval stages success. In fact, some polychaete species, such as *Sabella spallanzanii*, adopt in situ fertilization, a strategy which does not include the egg dispersal in the water column [8]; in these cases, mucus is the medium where eggs are released and even fertilized, as embedding eggs in the mucus enhances fertilization success [9]. In larvae, but also in adults, mucous formations have a function linked also to the capture of food, through agglutination and accumulation of particles [10,11]. In the family Sabellidae, the mucosal matrix is also involved in lubrification, gaseous exchange [10], and in cementing different substances in tube building, mixing feces, pseudofeces and sediment [12]. Many polychaetes are slow moving or sessile, thus they have developed a number of secreted compounds of the immune system [13,14]; among these immunomodulators, lysozyme-like activity was found in various polychaete species [15,16,17]. All these studies, however, concern the coelom, while Stabili et al. [9] examined the presence of lysozime-like activity in the mucus, which serves as a medium to exude antibacterial substances. Those polychaetes which have an infaunal mode of life, are often wrapped in a large amount of mucus [18]. Membranous mucoid secretions stabilize the burrow structure and dilute the bacterial numbers by a rapid turnover [19] and represent also a defense mechanism that protects the organism from environmental stresses including pollutants and reactive oxygen species (ROS) [20]. In the polychaete *Laeonereis acuta* the mucus release could be an antioxidant defense against environmental ROS [18] and the antioxidant competence is related to the carbohydrate content of its mucoproteins, which acts as radical scavenger. 

Taking into account the positive relationship between nutraceutical or functional food and human health, the research of bioactive molecules with antioxidant activity is interesting. In particular, natural antioxidant compounds function as health-protecting factors from free radicals and ROS actions [21]. In recent years, attention in natural antioxidants has improved because they are widely distributed and safer than synthetic antioxidants. In human disease the antioxidants may have a major therapeutic role determining also the regression of premalignant lesions and reducing their development into cancer [22]. Although attention has been mainly paid to terrestrial plants and microorganisms, marine invertebrates could also be investigated as potential sources of antioxidants [23]. In this framework, in the present study we investigated the widespread Mediterranean polychaete *Myxicola infundibulum,* which secretes a great amount of dense mucus producing a gelatinous envelope within which the worm lives [24]. Here we describe some chemical properties of *M. infundibulum* mucus such as elemental composition, the protein, lipid and carbohydrate content. In addition, the advanced analytical technique of multinuclear and multidimensional NMR spectroscopy was employed to further chemically characterize *M. infundibulum* mucus. Moreover, in order to highlight the potential of worm mucus as a potential prospect for drug discovery, the presence of bioactive compounds with antimicrobial and antioxidant activities was investigated.

## 2. Results

### 2.1. Analysis of Mucus Composition by NMR Spectroscopy

#### 2.1.1. Aqueous Extract 

**Figure 1:**
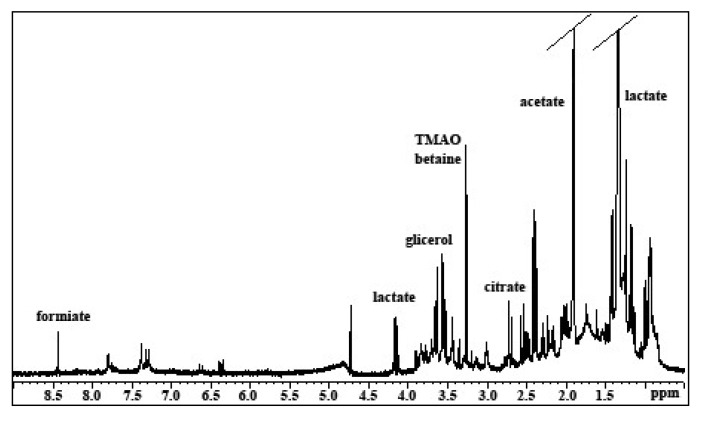
^1^H NMR spectrum with water pre-saturation in D_2_O of aqueous extract of *Myxicola infundibulum* mucus.

**Figure 2:**
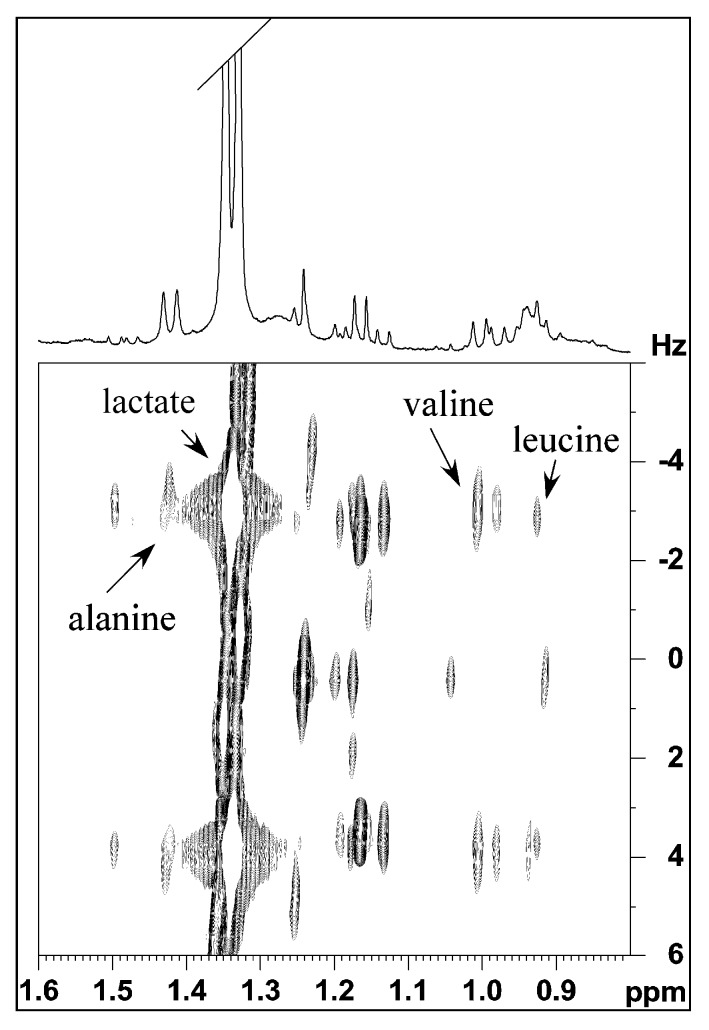
Expansion of ^1^H *J*res spectra with water pre-saturation of aqueous extract of *M. infundibulum* mucus.

By 1D and 2D ^1^H NMR spectroscopy of the aqueous extract (Figure 1) several amino acids and low molecular weight molecules were identified. In particular at low frequencies of the spectra there were the characteristic signals of the amino acids with aliphatic groups, such as leucine (two doublets at 0.92–0.94 ppm), valine (two doublets at 0.98–1.00 ppm) and alanine (a doublet at 1.49 ppm) (Figure 2). In addition, in this region more intense signals related to lactate (with a doublet at 1.33 ppm and a multiplet at 4.16 ppm, assigned respectively to the methyl and methine of lactate), and acetate (with a singlet at 1.91 ppm) were also observed.

**Figure 3:**
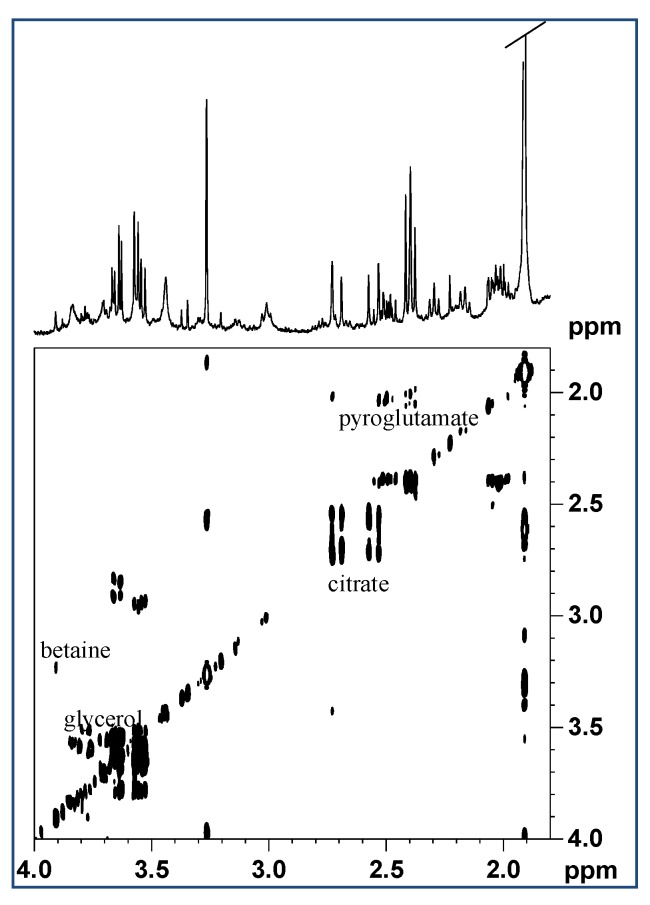
Expansion of ^1^H COSY spectra in D_2_O with water pre-saturation of aqueous extract of *M. infundibulum* mucus.

Additionally, the finding of citrate was of interest, with two doublets at 2.55 and 2.71 ppm (Figure 3). By the 2D 1H COSY spectra (Figure 3) the pattern signals with multiplets at 2.0, 2.4, and 2.5 ppm were assigned to pyroglutamate.

The pyroglutamic acid (2-pyrrolidone-5-carboxylic acid) (Figure 3) forms spontaneously from L-glutamate by cyclization of the internal amide, and its conversion speed is highly dependent on temperature and pH of the solution of L-glutamate. In addition, in 1D and 2D ^1^H NMR spectra, the multiplets at 3.55, 3.65, and 3.68 ppm, assigned to the free glycerol, and the singlet, at 3.27 ppm, attributed to tetramethylamminic group of Trimethylamine-N-oxide TMAO were identified. Under the latter signal the trimethylamminic group of betaine also resonates, showing a correlation cross peak with the singlet at 3.9 ppm, assigned to the –CH_2_- group of the same molecule (Figure 3).

#### 2.1.2. Lipidic Extract

**Figure 4:**
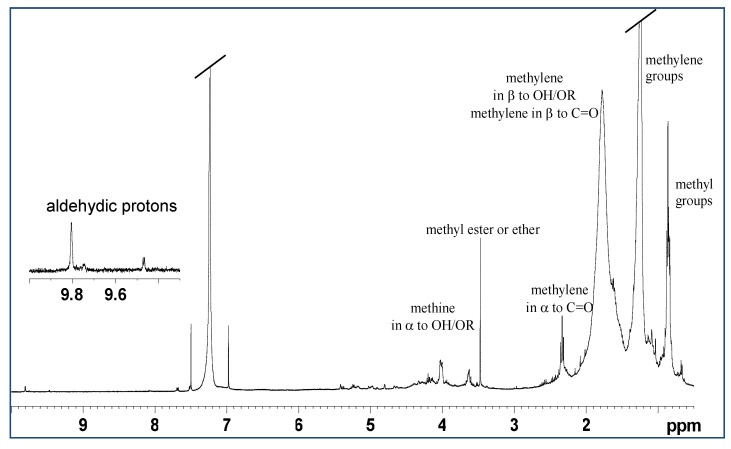
^1^H spectrum in CDCl_3_ of lipid extract of *M. infundibulum* mucus.

NMR spectroscopy showed a low content of lipids in the mucus of *M. infundibulum*. In the 1D ^1^H NMR spectra (Figure 4) and 2D ^1^H *J*res in CDCl_3_ a doublet at 0.84 ppm and a triplet at 0.86 ppm were observed. These two were assigned, respectively, to the methyls of branched chains (of the type iso, which have the branching on the penultimate carbon of the chain, (CH_3_)_2_-CH-, or anteiso with branching on the third last carbon, -CH-(CH_3_)-CH_2_CH_3_) and to the methyls of linear chains (CH_3_CH_2_CH_2_-). These signals, in the ^1^H ^13^C HSQC spectra, show correlation with carbons that resonate at about 14 ppm.

In the ^1^H NMR spectrum, the more intense multiplet at 1.24 ppm was assigned to the methylene groups of aliphatic chains, and another less intense broad multiplet at 1.77 ppm was attributed to the CH_2_ in β position in respect to carbonyl groups and to alcoholic groups, also etherified. In fact, in the ^1^H ^13^C HSQC experiment, these signals show correlation cross peaks with the carbons at 25 and 34 ppm. 

**Figure 5:**
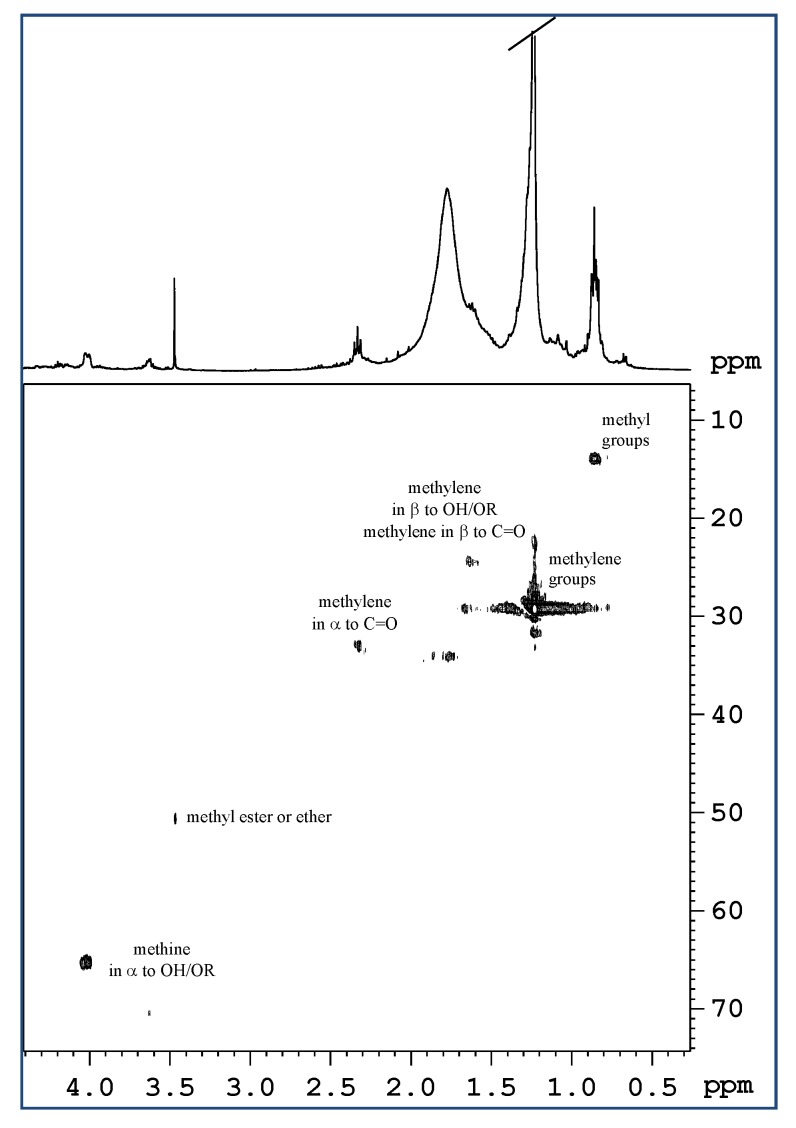
Expansion of ^1^H ^13^C HSQC spectra in CDCl_3_ of lipid extract of the mucus of *M. infundibulum*.

In the 1D ^1^H spectrum the triplet at 2.33 ppm was assigned to a CH_2_ group in α to carbonyl group. This signal in the ^1^H ^13^C HSQC exhibits a cross peak with a single carbon at 34 ppm. Since the intensity of this signal is very low compared to the signal at 1.77 ppm, assigned to CH_2_ in β position to the carbonyl group, it can be deduced that in this extract the presence of fatty acids is very scarce, and that lipids are preferentially alcohols or ethers (Figure 5). 

Confirming the fact that the lipids in this extract are of alcoholic origin, or otherwise hydroxylated, two rather intense multiplets at 3.64 and 4.03 ppm were observed. These two multiplets in the ^1^H ^13^C HSQC experiment show correlation cross peaks respectively with the carbons at 70 and 65 ppm.

These signals in the 2D homonuclear experiments (COSY and TOCSY) show couplings with aliphatic CH_2_ that resonate at lower frequencies and they have therefore been assigned to methine or methylene groups, linked to an alcoholic (OH) or ether (OR) function. It is interesting to note that the signal of vinyl groups is almost absent, except for a weak multiplet at 5.23 ppm.

### 2.2. Water and Inorganic Content

**Figure 6:**
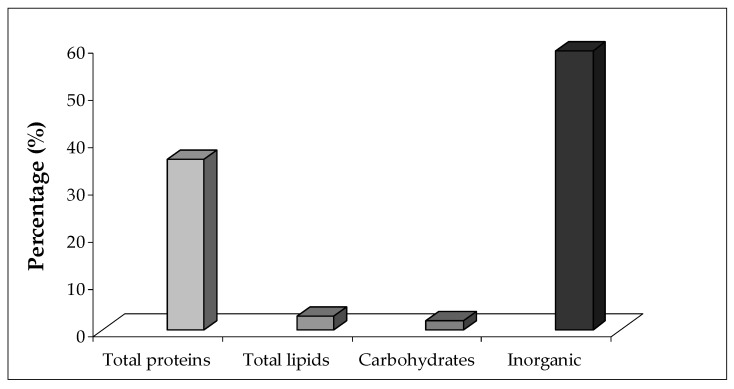
*M. infundibulum* mucus composition: organic (Total proteins, Total lipids, and Carbohydrates) and inorganic residuals.

*M. infundibulum* mucus contained about 94% ± 0.7% of water. Inorganic salts prevailed in the dried sample. A total of 59% ± 1.4% of the dry weight was indeed composed of inorganic elements, and the remainder of the dry weight (about 41%) was organic (Figure 6). In Table 1 the mean percentages of the elements detected in the mucus are reported. Data show that, in all samples, Cl and Na were the most abundant elements; by contrast C, Mg, and K ranged from 2.8% to 1.35% and Zn was present with the lowest percentage.

### 2.3. Protein, Lipid, and Carbohydrate Content

**Figure 7:**
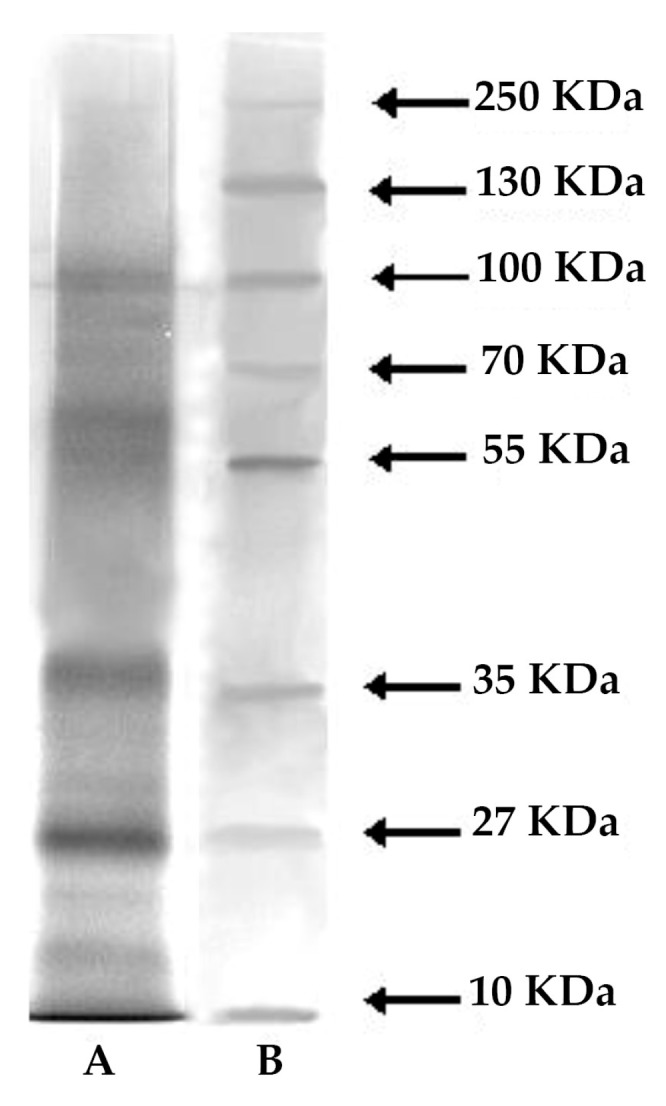
SDS-PAGE analysis and silver staining of *M. infundibulum* mucus. (**A**) Molecular weight standards furnished by Fermentas. Molecular weights (kDa) of standard proteins are on the left; (**B**) *M. infundibulum* total mucus.

Most of the organic residual of the mucus dry weight was composed of proteins (36% ± 2.3%) followed by lipids (2.9% ± 0.07%) and carbohydrates (2% ± 0.31%). The electrophoretic analysis revealed at least seven major protein bands, ranging from 12 to 200 kDa (Figure 7).

### 2.4. Antibacterial Activity

**Figure 8:**
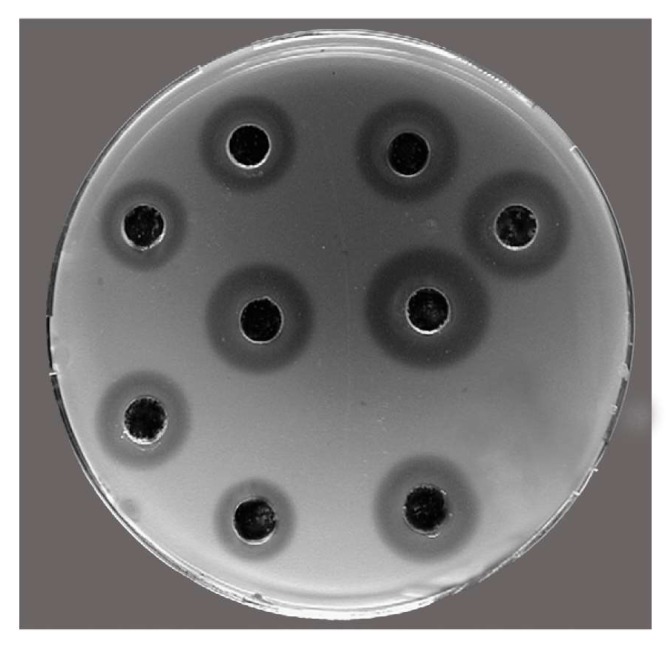
Antibacterial activity of *M. infundibulum* mucus against *Micrococcus luteus* determined by standard test on Petri dishes. Note cell wall lysis as the cleared zone surrounding wells on the test plate.

In Figure 8, the antibacterial lysozyme-like activity of *M. infundibulum* mucus is reported. By the standard assay the maximum diameter of lysis (8.3 ± 0.2 mm, corresponding to 1.14 mg mL^−1^ of hen egg-white lysozyme) was observed at an ionic strength of 0.175, pH = 6.0, and 37 °C.

### 2.5. Antioxidant Activity

Antioxidant activity of *M. infundibulum* mucus was detected by two methods based on two different chemical reactions in vitro: Trolox Equivalent Antioxidant Capacity (TEAC) assay and Oxygen Radical Absorbance Capacity (ORAC) assay. The antioxidant capacity of mucus measured by TEAC assay shows a value of 483.00 ± 79.22 nmolTE/mL while the same sample assayed by ORAC assay indicates a result of 276.26 ± 50.76 nmolTE/mL.

## 3. Discussion

In the present study we focused on the widespread Mediterrranean polychaete *M. infundibulum* which secretes a large amount of mucus which was chemically characterized and also investigated to highlight the presence of antimicrobial and antioxidant compounds with a view to potential biotechnological applications. 

From the obtained results some interesting issues can be inferred:

The mucus of *M. infundibulum* is mainly composed by water reaching a percentage of 94% ± 0.7%, accordingly with other studies on typical marine mucus [9] exerting a water content around 96–98%. The high percentage of inorganic material (about 59%) presumably results from dried salts left over when the seawater in a gel evaporates as already suggested for limpets and periwinkles mucus by Smith et al. [25] and Smith and Morin [26] which observed a similar proportion of inorganic material. A similar pattern was also recorded in the mucus of the polychaete *Sabella spallanzanii* having a water content of 96.2% ± 0.3% and a high percentage of inorganic material (about 65.2%). Chemical analyses revealed a low content of lipids (around 3%) and carbohydrates (around 2%).

NMR spectroscopy confirmed the scarcity of lipids in the mucus of *M. infundibulum* in accordance also with other studies reporting lipids as constituting 1–2% of mucus. Lipids play different roles in the mucus affecting the wettability, hydrophobicity and barrier functions, lubricating, surface tension, and rheological properties of the mucus layer [27]. Lipids also serve to prevent evaporation of the aqueous phase. From the lipid extract analysis of *M. infundibulum* mucus, the presence of fatty acids results scant, and lipids are preferentially represented by alcohols or ethers. On the other hand, from the analysis of the aqueous extract the presence of several amino acids was recorded demonstrating the protein nature of *M. infundibulum* mucus. In particular, valine, leucine, and alanine were the most abundant amino acids, as also found in the sea urchin *Paracentrotus lividus* footprint mucus [28]. NMR analysis of mucus worm detected also the presence of the pyroglutamic acid (2-pyrrolidone-5-carboxylic acid), which forms spontaneously from the amino acid L-glutamate (by cyclization of the internal amide), thus further confirming the protein nature of this matrix. 

In accordance with these NMR data on the protein nature of the mucus, the biochemical analyses revealed that the mucus of the investigated polychaete is mostly made up of proteins, constituting the main noticeable organic component (about 36% of total mucus dry matter). *M. infundibulum* mucus exerts an interesting protein pattern since from the electrophoretic analysis a complex of at least seven major proteins with a molecular weight ranging from 12 to 200 kDa was recorded. This evidence is in accordance with the general multi-protein nature of the adhesives present in other marine invertebrates including the mucus of the polychaete *S. spallanzanii* in which 10 major protein bands and six minor components were highlighted by SDS-PAGE analysis [29]. The footprint material of the sea urchin *Paracentrotus lividus* also have a multi-protein character since the soluble fraction consists of 13 protein bands with molecular weights comprised between 10 and 200 kDa [28]. Furthermore, evidences indicate that sea cucumbers and limpets also possess multi-protein complexes in non-permanent adhesives [30,31]. The lysozyme activity recorded in *M. infundibulum* mucus can be displayed by one of the seven major protein bands evidenced by the electrophoretic analysis. Interestingly, one of the “known proteins in the databases” described in a marine adhesive is a homolog of lysozyme in barnacle cement [32]. Lysozymes are enzymes acting as non-specific innate immunity molecules toward bacterial invasion since they produce the hydrolysis of the β 1–4 glycoside bond between N-acetylmuramic acid (NAM) and N-acetylglucosamine (NAG) damaging the bacterial cell wall [33]. Lysozymes can act via direct action or through their stimulatory effects on phagocytosis. *M. infundibulum* mucus possess a natural antibacterial lysozyme activity. Marine organisms, including invertebrates, have developed defense strategies towards several pathogens, mainly bacteria, living in their surrounding environment [34]. In particular, sessile invertebrates, including corals, sponges, and ascidians, have been extensively investigated for the production of an astonishing variety of antimicrobial compounds [35] useful to avoid bacterial surface attack and colonization [36,37]. By contrast, only a limited number of polychaete species have been studied in this respect [9,29,38,39]. Thus, the present paper represents an attempt to further investigate this zoological group from this point of view. The antibacterial lysozyme activity observed here represents a defense mechanism against bacterial infections for *M. infundibulum*. The lysozyme-like activity in the mucus evidenced here is noteworthy as a potential novel antibacterial compound since there is an increase of multidrug-resistant pathogens for humans. This emergence leads to the exploration of new antibacterial agents exerting deeply different modes of action than those of traditional antibiotics [40,41]. Among the most promising biocidal agents secreted by living organisms bacterial cell wall hydrolases (BCWH) are included and lysozyme was recently selected as a model protein [42,43]. These enzymes are environmentally benign and work more specifically than conventional organic biocides. Consequently, lysozyme from *M. infundibulum* mucus could be a great prospect in drug discovery as a new antimicrobial agent.

Interestingly, in the NMR spectrum, betaine, TMAO and glycerol were also found. These compounds are usually known to be organic osmolites, in fact they can be synthesized or gained from the environment in order to protect cells from osmotic stress, elevated temperature, and salinity [44]. It is well known that invertebrates use mucus as an external surface coating, useful to limit water loss. Presumably the presence of betaines in *M. infundibulum* mucus allows water retention, protecting the animals from the effects of dehydration. Glycerol is a low molecular weight carbohydrate. It is often accumulated in the cells as a remedy against osmotic stress, as quoted for instance by Oren [45] that reported high concentrations of glycerol in the cells of the microalga *Dunaliella salina*, a species that have to cope with extreme salinities. Besides being an osmoprotector, glycerol was also recognized by Muscatine [46] as an energy carrier. Hence, given the presence of these kinds of organic osmolites in *M infundibulum* mucus, it can be assumed that the abundant amount of this matrix around the polychaetes can hold an osmoprotective function for the worms. This polychaete species, living also in brackish water environments, may indeed have to cope with osmotic stress.

The characterization of *M. infundibulum* mucus is crucial since mucous matrices were recently taken into consideration for their potential applications. In fact, when particles are coated with mucus they get entangled together in a common mass, thus mucus naturally functions as a bio-adhesive. Although mucous matrices have received scarce attention with respect to permanent adhesives (characteristic of sessile organisms, such as mussels and barnacles), they are now starting to be considered for their efficiency in the adhesion phenomenon in aqueous media [28]. In this respect, investigations on the molecular mechanisms of non-permanent adhesives may lead to the design of new synthetic compounds, useful for wet environments, including surgery and dentistry, as well as the development of non-toxic coatings to contrast fouling organisms. 

*Myxicola infundibulum* mucus exerts a natural antioxidant activity. The assays for measuring antioxidant activity are classified on the basis of chemical reactions: methods based on hydrogen atom transfer (HAT) and methods based on electron transfer (ET) [47]. In this study the antioxidant capacity of *M. infundibulum* mucus was evaluated by means of two chemical in vitro assays: the Oxygen Radical Absorbance Capacity assay (ORAC), one of the HAT based assays and the Trolox Equivalent Antioxidant Capacity assay (TEAC), one of the ET based assays. Comparison of different analytical methods is strongly recommended for determining total antioxidant capacity of a crude sample [48,49]. The here obtained data indicate a higher antioxidant activity of mucus against 2,2’-Azinobis (3-ethylbenzothiazoline-6-sulfonic acid) diammonium salt (ABTS) radical cations measured by TEAC than against peroxyl radicals assayed by ORAC assay. These results suggest that in *M. infundibulum*, mucus antioxidant systems with different reaction mechanisms and kinetics are present. This is not surprising considering that as an invertebrate group, annelids lack adaptive immunity and consequently they have evolved sophisticated strategies of innate immunity mediated by both cellular and humoral components for defense against pathogens. In bivalves, for example, the cell-mediated immune response is the elimination of invaders by phagocytosis with an increase in oxygen consumption and an increase in ROS production, the so-called respiratory burst [50]. When high levels of ROS are generated, they can interact with lipids, nucleic acids and proteins, causing cell damage or death through oxidative stress [51]. In addition, considering that aquatic organisms have to cope with a great array of environmental pro-oxidants, it is evident the necessity of efficient antioxidant defense mechanisms. In several invertebrate and vertebrate aquatic species, including polychaetes, enzymatic and non-enzymatic antioxidant defenses, playing a role in inhibition of lipid peroxidation and metal ion chelation, have been already widely well recognized [18,52,53,54,55,56,57,58,59]. Abele-Oeschger and Oeschger [55] firstly suggested that non-conventional antioxidants play a role in polychaete defense and evidenced high antioxidant protection in the later developmental stages of the species *Phyllodoce mucosa*. Afterwards, da Rosa et al. [58] evidenced catalase-like activity in the mucus secreted by *Laeonereis acuta*. Information about the capability of the mucus to degrade environmental hydrogen peroxide (H_2_O_2_) was provided by da Rosa et al. [59] even though the antioxidant competence of mucus secretion toward other pro-oxidants was not taken into consideration. Later, Moraes et al. [18] observed antioxidant properties of the mucus produced by *L. acuta* and concluded that the mucus production is involved in the antioxidant defense system of the worms against environmental pro-oxidants through the interception or degradation of H_2_O_2_, peroxyl and hydroxyl radicals produced by fungal action in the sediment. Previous studies on marine organisms have shown that antioxidant activity is related to bioactive molecules like proteins, non-protein peptides, amino acids, or even polysaccharides involved in inhibition of lipid peroxidation and metal ion chelation [18,52,53]. On account of these studies, as well as our evidences for the antioxidant activity recorded in the mucus, it could be suggested that the activity is due to one or more active compounds evidenced from the biochemical and NMR analyses. It is well known that antioxidants are involved in the protection of the human body against damage by ROS [21] and currently there is a great interest towards novel sources of bioactive compounds playing a role in the prevention of oxidative stresses related to neurodegenerative diseases, cancer, and diabetes, and those that have antioxidant, immunomodulatory, analgesic, or anti-diabetic activities [60]. In this framework, the here evidenced antioxidant activity leads to conclude that the examined *M. infundibulum* mucus could be a new source of promising bioactive compounds for human welfare exploitable as pharmacological and cosmetic active ingredients. Obviously, further studies will be performed to assess whether the lysozyme type involved in the polychaete protection is the same in marine invertebrates as well as to estimate the antibacterial activity against other living microorganisms besides *Micrococcus luteus*. At this stage, we only focused on the occurrence of a lysozyme-like and antioxidant activity in the whole mucus. In a previous work [61] we investigated *M. infundibulum* mucus highlighting its role as both bacterial control and a bacterial home site, in the present manuscript we deeply investigated this matrix and focused on the potential prospect of worm mucus for drug discovery by adding several additional analyses and information. However, to further clarify the mechanisms related to *M. infundibulum* defenses, the isolation, purification, and quantification of the effectors involved in such activities are needed and will be carried out in the near future 

In conclusion, considering the scant knowledge of the structure and biochemistry of the marine invertebrate mucous matrices, the present study contributes to this topic demonstrating that *M. infundibulum* mucus represents a potential marine accessible and renewable resource that could require biotechnological exploitation for numerous possible applications for drug discovery, useful for health promotion, and disease prevention.

## 4. Materials and Methods 

### 4.1. Animals and Sample Preparation 

One hundred adult specimens of *M. infundibulum* were sampled by using SCUBA equipment in the Ionian Sea (Mar Grande, Taranto, Italy). After collections *M. infundibulum* specimens were transferred into a cooling container at approximately 5 °C, and rapidly transported to the laboratory within 4 h from collection for further processing. In the laboratory, the secretion of the mucus was stimulated by placing each individual of *M infundibulum*, removed from the tube, in a Petri dish and keeping them for 30 min. In these conditions, animals spontaneously released mucus. Secreted mucus was collected and centrifuged at 12,000× *g* for 30 min at 4 °C. A previous work [10] showed that the protein content of the mucus varies between individuals. To avoid the introduction of this variable, in the present work the mucus of the whole group of 100 individuals was pooled into five samples (each pool collected from 20 polychaetes) which were stored at −80 °C until use and then employed for the NMR analysis, biochemical analyses, and the determination of the antibacterial lysozyme-like and antioxidant activity.

### 4.2. NMR Spectroscopy

The mucus samples of *M. infundibulum* were characterized by ^1^H and ^13^C 1D and 2D NMR spectroscopy with the same methodology already reported in Stabili et al. [61]. 1D ^1^H and ^13^C, 2D ^1^H *J*res, ^1^H COSY, ^1^H–^13^C HSQC and ^1^H–^13^C HMBC spectra were recorded at 298.15 K on a Bruker Avance III 400 MHz spectrometer (Bruker, Milan, Italy). CDCl_3_ was used as solvent. Chemical shift was referred to TMS by the residual protic solvent peaks as internal (^1^H = 7.24 ppm; ^13^C = 77.0 ppm). The acquisition of high resolution ^13^C NMR spectra was performed semi-quantitatively [62], with high number scans and short relaxation times to obtain sufficient S/N ratio to calculate the integrals. The considered parameters were: 64 K data points, spectral width of 20161.291 Hz, 16 K scans with a 0.5 s repetition delay and 60° as ^13^C excitation pulse. The software Topspin 2.1 (Bruker Biospin, Milan, Italy) was used for the acquisition and processing of spectra. Resonances of fatty acids and metabolites were provided according to literature data [63,64,65].

### 4.3. Water, Lipid, Protein, and Carbohydrate Content 

For water content measurement, the wet weights of mucus (three replicates for each of the five samples) were measured on an analytical balance. They were then dehydrated in a SpeedVac (Thermo Fisher Scientific, Waltham, MA, USA) and their dry weight was measured. Total lipids from each mucus sample were extracted according to the method of Folch et al. [66]. The mucus was homogenized with chloroform/methanol (2:1) to a final volume 20 times the volume of the mucus sample. After centrifugation and siphoning of the upper phase, the lower chloroform phase contained the lipids. Total lipid content was determined by the colorimetric enzymatic method [67] using commercial kit (FAR—Verona, Italy). The protein concentration of each mucus sample was measured using the Bradford assay [68] with bovine serum albumin (BIO-RAD, Milan, Italy) as standard. The carbohydrate concentration of the mucus was assayed using the method described by Dubois et al. [69] and Kennedy and Pagliuca [70]. The assay was calibrated with known amounts of D-glucose. 

### 4.4. Electrophoresis

Mucus samples were analyzed by SDS-polyacrylamide gel electrophoresis (SDS-PAGE). They were run on discontinuous gels, based on the method of Laemmli [71] and the detailed protocols of Hames [72]. The gels contained 10% of acrylamide, and were 8 × 9 cm by 1.0 mm thick. The migration buffer consisted of 25 mM Tris, 192 mM glycine, pH 8.5. After migration, gels were stained using Silver Stain kit (Sigma, St. Louis, MO, USA). Molecular standards (PageRuler™ Prestained Protein Ladder range 10–250 kDa, Fermentas, Waltham, MA, USA) consisted in a mixture of eight recombinant, highly purified, colored proteins with apparent molecular weights of 10 to 250 KDa.

### 4.5. Antibacterial Activity

Lysozyme-like activity was evaluated by using inoculated Petri dishes as standard assay [73]. Briefly, 700 mL of 5 mg/mL of dried *M. luteus* cell walls (Sigma) was diluted in 7 mL of 0.05 M PB-agarose (1.2%) (pH 5.2) and then spread on a Petri dish. After agarose gel solidification wells with a diameter of 6.3 mm diameter were sunk and filled with 30 μL of the sample (three replicates for each of the five samples). After overnight incubation at 37 °C the diameter of the cleared zone, due to the lysis of bacterial cell walls, of five replicates was measured. The recorded diameters were then compared with those of a reference sample represented by hen egg-white lysozyme (Merck, Darmstadt, Germany) used at a concentration ranging from 0.2 to 1.5 mg/mL and producing diameters of lysis comprised between 1.5 and 10.5 mm.

### 4.6. Antioxidant Activity 

#### 4.6.1. Oxygen Radical Absorbance Capacity Assay (ORAC)

For ORAC the method of Davalos et al. [74] was used; mucus (three replicates for each of the five samples) was diluted with 75 mM phosphate buffer (pH 7.4). The assay was carried out in 96-well plates (Greiner-Bio One, Frickenhausen, Germany) using an Infinite200Pro plate reader (Tecan, Männedorf, Switzerland). To each well 20 µL of samples and 120 µL of fluorescein (FL; 70 nM final concentration) were added, and the plate was incubated at 37 °C for 15 min. Finally, the AAPH (60 µL; 12 mM final concentration) was added to each well and fluorescence intensity was estimated every minute for 80 min using an excitation wavelength of 485 nm and an emission wavelength of 535 nm. A standard curve was constructed using 6-hydroxy-2,5,7,8-tetramethylchroman-2-carboxylic acid (Trolox, Sigma-Aldrich,1.5–10.5 µM). A blank (fluorescein + AAPH) using phosphate buffer instead of the sample was carried out in each assay. Results were determined computing the difference in area under the curve between the control and the sample by using Magellan v7.2 software (Tecan, Switzerland) and expressing the result as nmoles of Trolox equivalents (TE) per mL of mucus. All the reaction mixtures were prepared in triplicate and at least three independent assays were performed for each sample. 

#### 4.6.2. Trolox Equivalent Antioxidant Capacity Assay (TEAC)

The TEAC assay was performed as described by Re et al. [75] with minor modifications to adapt the assay to a microplate reader [62]. 2,2’-Azinobis (3-ethylbenzothiazoline-6-sulfonic acid) diammonium salt (ABTS, Sigma-Aldrich) radical cations were obtained by mixing the same volume of an aqueous solution of potassium persulfate 2.45 mM (final concentration) and an aqueous solution of ABTS 7 mM (final concentration) and allow to stand in the dark at room temperature for 12–16 h. The obtained ABTS radical cation solution was diluted in PBS (pH 7.4) to obtain an absorbance of 0.40 at 734 nm ± 0.02; this value was adopted to obtain about 80% of maximum inhibition of the blank absorbance using the highest concentration of the Trolox standard curve (0–16 µM). In each well of a 96 well-plate (Costar, MERCK, Darmstadt, Germany), 200 µL of diluted ABTS radical cation and 10 µL of Trolox standard or mucus diluted in PBS were added, and the absorbance at 734 nm was read 6 min after initial mixing, using an Infinite200Pro plate reader (Tecan, Männedorf, Swizerland). Each of the five mucus samples were assayed in least at three separate dilutions and in triplicate. The percentage inhibition of absorbance at 734 nm was calculated and compared to the Trolox standard curve: the TEAC value was expressed as Trolox equivalent (in nmols) per mL of mucus, using Magellan v7.2 software (Tecan, Männedorf, Switzerland).

## Figures and Tables

**Table 1:** Elements detected in mucus sample of *M. infundibulum.*

Element	Content %
C	2.34 ± 0.02
H	1.79 ± 0.11
N	0.66 ± 0.02
Fe	absent
Ca	1.08 ± 0.02
Mg	2.82 ± 0.02
Zn	0.04 ± 0.005
Cu	absent
K	1.35 ±0.02
Na	14.87 ± 0.10
Cl	34.13 ± 0.15
P	absent
Sn	absent
Se	absent
